# Reduction of PINK1 or DJ‐1 impair mitochondrial motility in neurites and alter ER‐mitochondria contacts

**DOI:** 10.1111/jcmm.13815

**Published:** 2018-08-22

**Authors:** Cristina Parrado‐Fernández, Bernadette Schneider, Maria Ankarcrona, Melissa M. Conti, Mark R. Cookson, Miia Kivipelto, Ángel Cedazo‐Mínguez, Anna Sandebring‐Matton

**Affiliations:** ^1^ Division of Neurogeriatrics Department of Neurobiology, Care Sciences and Society Center for Alzheimer Research Karolinska Institutet Stockholm Sweden; ^2^ Laboratory of Neurogenetics National Institute on Aging/NIH Bethesda Maryland; ^3^ Division of Clinical Geriatrics Center for Alzheimer Research NVS Karolinska Institutet Stockholm Sweden; ^4^ Aging Research Center Karolinska Institutet‐Stockholm University Stockholm Sweden; ^5^ Research & Development Unit Stockholms Sjukhem Stockholm Sweden

**Keywords:** ER‐mitochondrial contacts, mitochondrial motility, Parkinsonism, PINK1

## Abstract

Subcellular distribution of mitochondria in neurons is crucial for meeting the energetic demands, as well as the necessity to buffer Ca^2+^ within the axon, dendrites and synapses. Mitochondrial impairment is an important feature of Parkinson disease (PD), in which both familial parkinsonism genes *DJ‐1* and *PINK1* have a great impact on mitochondrial function. We used differentiated human dopaminergic neuroblastoma cell lines with stable PINK1 or DJ‐1 knockdown to study live motility of mitochondria in neurites. The frequency of anterograde and retrograde mitochondrial motility was decreased in PINK1 knockdown cells and the frequency of total mitochondrial motility events was reduced in both cell lines. However, neither the distribution nor the size of mitochondria in the neurites differed from the control cells even after downregulation of the mitochondrial fission protein, Drp1. Furthermore, mitochondria from PINK1 knockdown cells, in which motility was most impaired, had increased levels of GSK3βSer9 and higher release of mitochondrial Ca^2+^ when exposed to CCCP‐induced mitochondrial uncoupling. Further analysis of the ER‐mitochondria contacts involved in Ca^2+^ shuttling showed that PINK1 knockdown cells had reduced contacts between the two organelles. Our results give new insight on how PINK1 and DJ‐1 influence mitochondria, thus providing clues to novel PD therapies.

## 1 INTRODUCTION

Parkinson disease (PD) is an age‐associated progressive neurodegenerative disorder characterized by dopaminergic neuronal cell death in the substantia nigra. Although disease progression involves aberrant mitochondrial functionality,^1^, ^2^, ^3^ the underlying molecular mechanisms are not completely understood. The outcome from malfunctioning mitochondria in dopaminergic neurons involves disturbance in oxidative phosphorylation, loss of mitochondrial membrane potential and incomplete Ca^2+^ buffering capacity, resulting in bio‐energetic failure and neuronal dysfunction.^4^ Furthermore, mitochondria also contain proteins regulating multiple intracellular signalling pathways,^5^ therefore maintaining healthy mitochondria is crucial for the survival of the whole cell. Interestingly, several of the familial parkinsonism causative genes exhibit functions of importance for mitochondrial status.^6^ Notably, mutations in PTEN‐induced Kinase‐1 (PINK1) and parkin are the most common cause of autosomal recessive, early‐onset familial form of PD and have been found to lead to dramatic effects on mitochondrial morphology, quality control and functionality in both human and diverse model organisms.^7^, ^8^, ^9^, ^10^

PINK1 has a mitochondrial targeting peptide, while the mature protein has been shown to localize both to mitochondria and cytosol.^11^, ^12^, ^13^, ^14^ On the outer mitochondrial membrane, PINK1 kinase activity recruits Parkin from the cytosol and triggers the autophagy of damaged mitochondria.^15^, ^16^ PINK1 can also phosphorylate motor‐adaptor protein Miro1 and thereby modulate mitochondrial motility in a Parkin‐dependent manner.^15^, ^17^, ^18^, ^19^ Because Miro1 contains Ca^2+^‐sensing EF hand domains, local Ca^2+^ concentrations regulate mitochondrial motility. Mitochondria buffer Ca^2+^ and exchange Ca^2+^ content with the endoplasmic reticulum (ER) in regions known as mitochondrial‐associated ER membranes (MAM), which influences mitochondrial motility and downstream mechanisms such as synaptic transmission and mitophagy.^20^, ^21^ Indeed, Gelmetti et al^16^ have recently demonstrated in human cell models that under mitophagic stimuli, PINK1 relocalize at MAM and may promote ER‐mitochondria tethering. Mutations in the DJ‐1 gene are another cause to autosomal recessive inherited parkinsonism.^22^ DJ‐1 is an ubiquitously expressed redox protein involved in several cellular signalling pathways and transcriptional regulation.^23^, ^24^, ^25^, ^26^, ^27^ Exposure to mitochondrial toxins or oxidative stress triggers the translocation of DJ‐1 protein to mitochondria^28^, ^29^ activating different neuroprotective mechanisms.^28^, ^30^, ^31^ Furthermore, overexpression of Parkin^32^ or DJ‐1^33^ showed an increased ER‐mitochondria interaction,^20^ whereas DJ‐1 loss‐of‐function induces ER‐mitochondria Ca^2+^ transfer, affects ER‐mitochondria tethering^33^ and interferes with mitochondrial fusion and fission.^34^

Mitofusins (Mfn) and optic atrophy 1 (Opa1) regulate fusion of the outer and inner mitochondrial membranes (OMM, IMM), respectively, whereas Dynamin‐related protein 1 (Drp1) and mitochondrial fission 1 protein (Fis1) coordinates mitochondrial division (reviewed in Ref. 35). We and others have shown that a decreased rate of fusion in human PINK1 and DJ‐1 deficient cells can be reversed by Drp1 downregulation which highlights the relevance of mitochondrial dynamics for PD neuropathology.^25^, ^36^, ^37^, ^38^ There is no doubt that PINK1 and DJ‐1 regulate cellular mechanisms that are crucial for cell survival, still the field have not yet reached a consensus on what are the pathological mechanisms from PINK1 or DJ1 loss‐of‐function in human patients. It appears that PINK1 is involved in multiple functions and some of the mitochondrial features may differ according to the cell type or model organisms used.^39^ Mitochondrial motility is an important feature for neurons and especially in the highly branched and energy demanding neurons of the substantia nigra.^50^ In the same manner as for the role of PINK1 in mitophagy and mitochondrial dynamics, previous reports on the role of PINK1 in mitochondrial motility show conflicting results depending on the model used.^17^, ^18^, ^41^ Our aim with the current study is to explore the effects from PINK1 or DJ‐1 knockdown on mitochondrial motility in a human RA/BDNF differentiated cell model. By confocal live imaging, we quantified the frequency of motility in fluorescently labelled mitochondria and evaluated the interplay of downregulated Drp1 with stable knockdown of PINK1 or DJ‐1. Furthermore, we aimed at characterizing whether loss of PINK1 could be directly implicated in the impact of mitochondrial Ca^2+^ buffering and ER‐mitochondria interactions as upstream factors of the reduced frequency in mitochondrial motility.

## 2 MATERIAL AND METHODS

### 2.1 Cell culture, plasmids and transfection

Clonal M17 cell lines stably expressing different control, PINK1 and DJ1 shRNA constructs were manufactured and cultured as described previously.^26^, ^38^ Differentiation of cells was by exposure to 10 μmol/L retinoic acid (RA, Sigma‐Aldrich, MO, USA) for 7 days in serum‐containing media followed by 7 day exposure to 25 ng/mL of brain derived neurotrophic factor (BDNF, Sigma‐Aldrich, MO, USA) in serum deprived media. Control (GFP) siRNA and DRP1 siRNA expression vectors were generous gifts from Dr Richard Youle (NINDS, Bethesda, MD) and were transfected using Lipofectamine 2000 (Invitrogen, Carlsbad, CA) according to manufacturer's instructions.

### 2.2 Immunocytochemistry

Cells were differentiated on Matrigel (BD Biosciences, San Jose, CA, USA)‐coated glass cover slips and stained with Mitotracker Orange (Invitrogen) according to manufacturer's instructions followed by fixation with ice‐cold 99.8% methanol (Sigma‐Aldrich) on ice. Cells were permeabilized with 0.1% Triton X‐100 in 1× PBS for 10 minutes and then blocked in 5% BSA and 2% goat serum (Sigma‐Aldrich) diluted in 0.1% Triton X‐100 in 1× PBS for 1 hour at room temperature. Probing with primary β‐actin antibody (1:500; Sigma‐Aldrich) was over‐night at +4°C. After repeated washing, cells were incubated for 1 hour with secondary AlexaFluor488 conjugated anti‐rabbit IgG and DAPI (1:500 and 1:1000 respectively, Invitrogen) followed by repeated washing steps and mounting on glass slides with ProLong antifade mounting medium (Invitrogen).

Images were captured using an inverted Laser Scanning Microscope (LSM 510 META; Zeiss, Oberkochen, Germany).

### 2.3 Subcellular fractionation

Cells were lysed in lysis buffer (20 mmol/L Tris‐HCl, 137 mmol/L NaCl, 2 mmol/L EDTA, 2 mmol/L EGTA 2% Nonidet P‐40, 2% Triton‐X100 supplemented with protease and phosphatase inhibitor cocktails from Sigma‐Aldrich). Mitochondrial fractions from undifferentiated cells were purified using a mitochondria isolation kit for cultured cells (Pierce, Rockford, IL) according to the manufacturer's instructions and were subsequently suspended in ice‐cold lysis buffer followed by 1 second ×10 sonication pulses at 14 μm.

The subcellular fractionation from mouse brain tissue into MAM, plasma membrane (PM), ER, and pure mitochondria fraction was performed by a method established previously in our lab.^42^

### 2.4 Western blotting

Protein extraction was quantified using the BCA protein assay kit (Pierce). Equivalent amounts of protein were separated using 10% acrylamide gels and were transferred to a nitrocellulose membrane (Whatman, GE Healthcare Group, Uppsala, Sweden).

Primary antibodies were used at the following concentrations: GSK3β‐Phospho‐Serine9, 1:1000 (Cell Signaling, Danvers, MA), TOM20, 1:1000 (Santa Cruz Biotech, Dallas, TX, USA), β‐tubulin, 1:2000 (Abcam, Cambridge, UK), DJ‐1, 1:1000 (Stressgen, San Diego, CA), PINK1, 1:1000 (Abcam), Miro1, 1:1000 (Abcam), GAPDH, 1:1000 (Sigma‐Aldrich, MO, USA). The secondary antibodies anti‐rabbit or anti‐mouse horseradish peroxidase‐linked (GE Healthcare, Marlborough, MA, USA) were used at 1:2000 dilution for 1 hour in room temperature. Detection was made by the ECL method and exposure to Hyper film MP (GE Healthcare). For Miro1 and GAPDH, western blotting and detection were according to protocol set forth by LI‐COR Near Infrared Western Blot detection. Primary antibodies were incubated over night and secondary for 1 hour at RT. Blots were imaged using an Odyssey CLx system and quantification was carried out using instrument's Image Studio software (Cambridge, UK).

### 2.5 Analysis of mitochondrial motility and data acquisition

Cells were differentiated in glass bottom microwell dishes coated with Matrigel. Time‐lapse imaging was performed at 37°C and 5% CO_2_ using an inverted Laser Scanning Microscope (LSM 510 META; Zeiss). One microgram of Mito‐DsRed2 (Clontech Laboratories, Mountain View, CA, USA) DNA was co‐transfected with 1 μg of either control siRNA or Drp1 siRNA constructs in order to visualize mitochondria in the differentiated cells. After 72 hours, time‐lapse images were collected ~512 × 512 pixel resolution (8 bit) and cells were imaged for 20 minutes using an interval of 5 seconds. Kymographs from straightened axons^43^ were acquired using ImageJ software (Rasband, W.S., ImageJ, U. S. National Institutes of Health, Bethesda, Maryland, USA, https://imagej.nih.gov/ij/, 1997‐2016) and were used to manually trace and count the frequency of anterograde, retrograde and total (anterograde + retrograde) motility events. Mitochondria switching direction were counted for each motile event, yet had to displace at least 5 μm to be counted. From the same sets of images, we counted the number of mitochondria in each neurite for estimating mitochondrial density, normalized to the neurite length (in μm) and also measured the individual mitochondrial length using the LSM 510 software (Zeiss, Oberkochen, Germany).

### 2.6 Measurement of cytosolic Ca^2+^ concentration

Cytosolic free [Ca^2+^] ([Ca^2+^]_i_) was measured in undifferentiated cells using the fluorescent ratiometric Ca^2+^ indicator indo‐1. Cells were cultured on glass bottom microwell dishes (MatTek Corporation, Ashland, MA, USA) coated with Matrigel (BD Biosciences) and loaded with indo‐1 by exposing them to indo‐1‐AM (8.3 μmol/L, Invitrogen, Carlsbad, CA, USA) for 60 minutes followed by at least 15 minutes of washing. Indo‐1 was excited with light at 360 nm, and light emission at 405 ± 5 and 495 ± 5 nm was measured with two photomultiplier tubes (PTI, Wedel, Germany). The ratio of the light emission at 405 nm to that at 495 nm represents [Ca^2+^]_i_.

### 2.7 Electron microscopy

Undifferentiated M17 cells from each line were fixed in 2.5% glutaraldehyde in 0.1 mol/L phosphate buffer, pH 7.4 at room temperature for 30 min. The cells were scraped off and transferred to eppendorf tubes and further fixed overnight in the refrigerator. Cells were then rinsed in 0.1 mol/L phosphate buffer and centrifuged. The pellets were postfixed in 2% osmium tetroxide (TAAB, Berks, UK) in 0.1 mol/L phosphate buffer, pH 7.4 at 4°C for 2 hours, dehydrated in ethanol followed by acetone and embedded in LX‐112 (Ladd, Burlington, VT, USA). Ultrathin sections (approximately 50‐60 nm) were cut by a Leica ultracut UCT (Leica, Wien, Austria). Sections were contrasted with uranyl acetate followed by lead citrate and examined in a Hitachi HT 7700 (Tokyo, Japan) at 80 kV. Digital images were taken using a Veleta camera (Olympus Soft Imaging Solutions, GmbH, Münster, Germany). Number and length of ER‐mitochondria contacts as well as mitochondrial length were quantified by a blinded person using Image J where contents from 120 images were quantified from two independent experiments. ER‐mitochondria contacts was exclusively measured in mitochondria showing the whole organelle. In the analysis, ER‐mitochondria contacts was considered to be formed when the distance between the two membranes was ≤30 nm.

### 2.8 Statistical analyses

Non‐parametric group comparisons of means were performed using Mann‐Whitney test, as specified in the legend of each figure. The null hypothesis was rejected at *P*‐value < 0.05. All statistical computations were carried out using GraphPad Prism Software, Version 5.0 (http://www.graphpad.com/prism/Prism.htm). Data values are expressed as mean ± standard error mean.

## 3 RESULTS

### 3.1 Downregulation of PINK1 or DJ‐1 does not affect neuritic length nor mitochondrial features in differentiated cells

**Figure 1:**
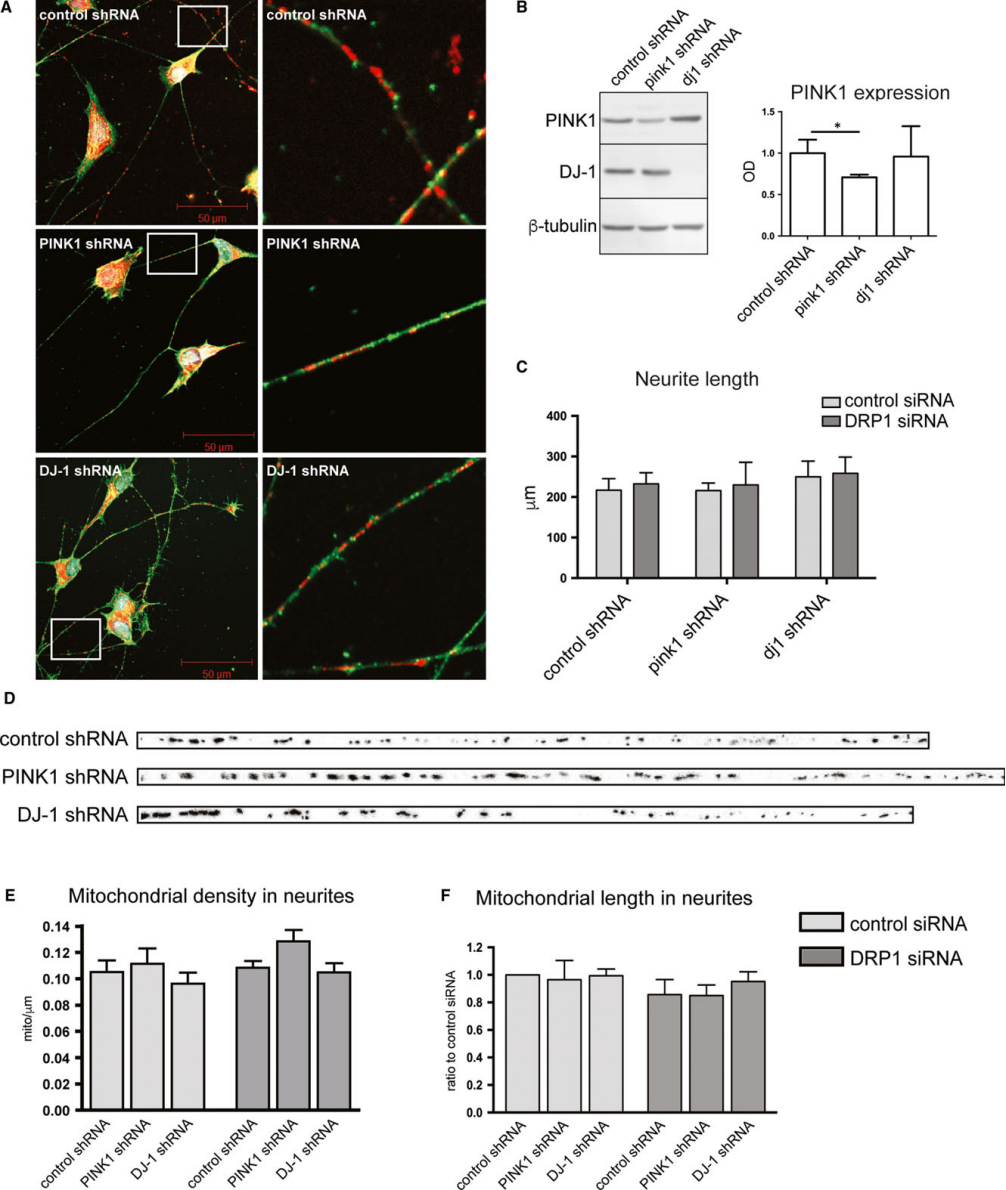
PINK1 or DJ‐1 knockdown does not obstruct differentiation in M17 neuroblastoma cells. Differentiated M17 neuroblastoma control, PINK1 and DJ‐1 shRNA expressing cells stained with mitotracker (red), β‐actin (green), and DAPI (blue) reveal a neuronal like morphology with extended neurites containing migrating mitochondria (A). Stable shRNA knockdown resulted in a 30% decrease of PINK1 protein as quantified in the right panel, and a full knockdown of DJ‐1 (B). Neurite length was not affected by PINK1 or DJ1 expression or by transient siRNA knockdown (C). Representative image of neurites in each RA/BNDF‐differentiated cell line with mitochondria in black (D). Quantification of the number of mitochondria normalized to μm neurite length from five independent experiments showed that mitochondrial distribution was equal between cell lines and was not affected by Drp1 siRNA transfection (E). Mitochondrial length expressed as ratio to control shRNA transfected with control siRNA showed no differences between cell lines (n = 3) (F). Data expressed as means ± SEM using Mann‐Whitney test (*P < 0.05)

We first aimed at evaluating our RA/BDNF differentiated human cell model in order to see if PINK1 or DJ‐1 depletion and transient reduction of Drp1 would have an impact on neurite length, mitochondrial density or mitochondrial length. Neuroblastoma cell lines stably expressing control, PINK1 or DJ‐1 shRNA were differentiated and then stained with Mitotracker (green) and β‐actin (red) to visualize mitochondria and cytoskeleton by confocal microscopy. All cell lines were able to form similar neuronal‐like morphology with extended neurites without PINK1 or DJ‐1 visibly interfering in the differentiation process (Figure 1A,C). Stable PINK1 shRNA expression result in 16% mRNA reduction in M17 cells^38^ followed by 30% of protein reduction, whereas DJ‐1 shRNA yielded no visible protein band (Figure 1B). We previously demonstrated that the knockdown of Drp1 prevents the mitochondrial fragmentation associated with loss of PINK1 expression.^38^ Here, we performed co‐transfections with Mito‐DsRed2 in combination with either control siRNA or Drp1 siRNA in differentiated cells to determine the length of mitochondria along the neurites. Figure 1D shows representative pictures of neurites from three independent experiments. The fluorescently labeled mitochondria (black spheres) were counted and measured for each neurite and normalized to the neuritic length in μm. Neither PINK1 or DJ‐1 stable knockdown or transient Drp1 silencing alter mitochondrial density along the neurites (Figure 1E). Mitochondrial length in neurites was also found to be similar between cell lines and was not affected upon Drp1 downregulation (Figure 1F).

### 3.2 The frequency of mitochondrial motility was reduced in human PINK1 and DJ‐1 knockdown cells

Recent studies show that PINK1 has an important role in mitochondrial motility in neurons.^17^, ^18^, ^41^, ^44^ However, to our knowledge, mitochondrial motility have not been studied in human PINK1 and DJ‐1 knockdown cells exposed to Drp1 siRNA. Since we and others have previously shown that either PINK1 and DJ‐1 depletion in a similar manner disrupt mitochondrial morphology that could be rescued by Drp1 siRNA,^25^ we were interested in investigating mitochondrial motility in the same cell model differentiated with RA/BDNF.

**Figure 2:**
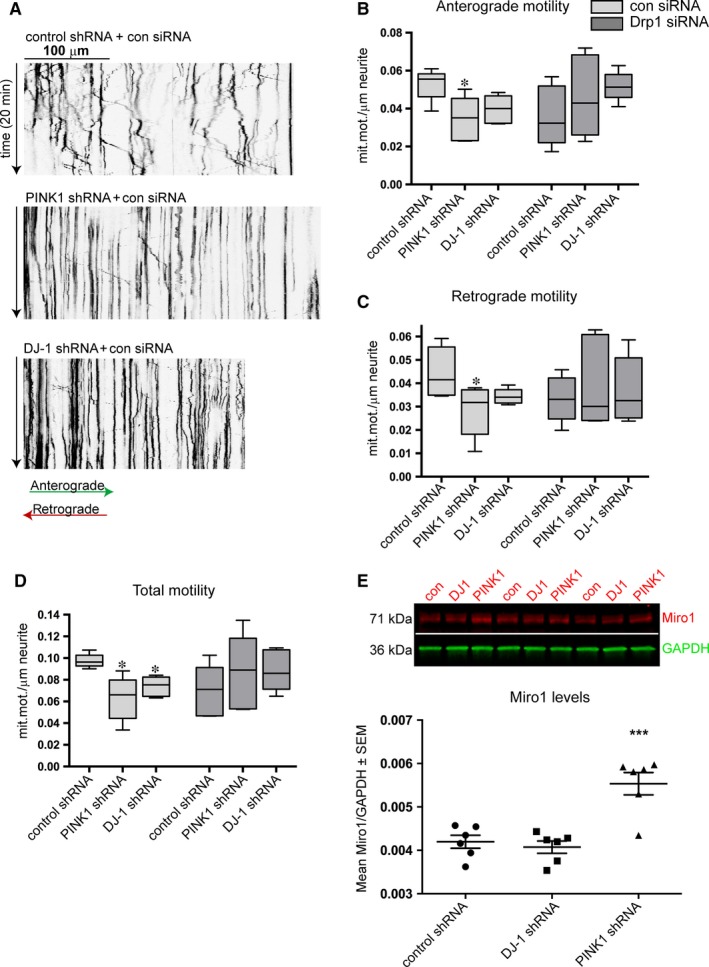
Expression of PINK1 and DJ‐1 is important for mitochondrial motility. Representative kymographs from 20 min live imaging from each RA/BNDF‐differentiated cell line Black lines representing mitochondria either moving anterogradely (lines towards the right‐hand side) retrogradely (towards the left‐hand side) or immobile (straight line) (A). The number of anterograde motility events was decreased in PINK1 shRNA cells compared to control shRNA when transfected with control siRNA for 72 h (B). Mitochondrial retrograde motility was decreased in PINK1 knockdown cells (C) yet the total motility events (anterograde + retrograde) was found to be significantly reduced by both PINK1 and DJ‐1 knockdown using Mann‐Whitney test (*P < 0.05) (D). Data are acquired from five independent experiments performed in triplicates and is expressed as means ± SEM. Levels of Miro1 and GAPDH were analysed with Western blotting and quantified as visualized in the graph. PINK1 depleted cells had higher levels of Miro1 compared to control cells and DJ‐1 knockdown (E, ***P < 0.001)

Differentiated cells were co‐transfected with mitochondrial‐targeted DsRed2 and control siRNA or Drp1 siRNA for 72 hours and then imaged every 5 seconds for 20 minutes. Blinded quantification of the resulting kymographs (Figure 2A) revealed that both anterograde and retrograde mitochondrial transport were significantly reduced in PINK1 shRNA cells compared to the control (*P* < 0.05; Figure 2B,C). DJ‐1 knockdown cells displayed a reduced number of anterograde and retrograde motile events near significance (*P* = 0.057) compared to control cells (Figure 2B,C). The total motility (anterograde + retrograde) eventually showed a significant reduction in both PINK1 and DJ‐1 knockdown cells (*P* < 0.05 for both; Figure 2D). Drp1 downregulation did not lead to changes in anterograde and/or retrograde motility of PINK1 and DJ‐1 knockdown cells. Since PINK1 is known to target the mitochondrial anchoring protein Miro1 for degradation by parkin,^18^ we next analysed the levels of Miro1 in our cells. PINK1 depletion resulted in a significant increase of Miro1 levels in our model (*P* < 0.001; Figure 2E).

### 3.3 Level of GSK‐3βSer9 is increased in the mitochondria of PINK1 deficient cells

**Figure 3:**
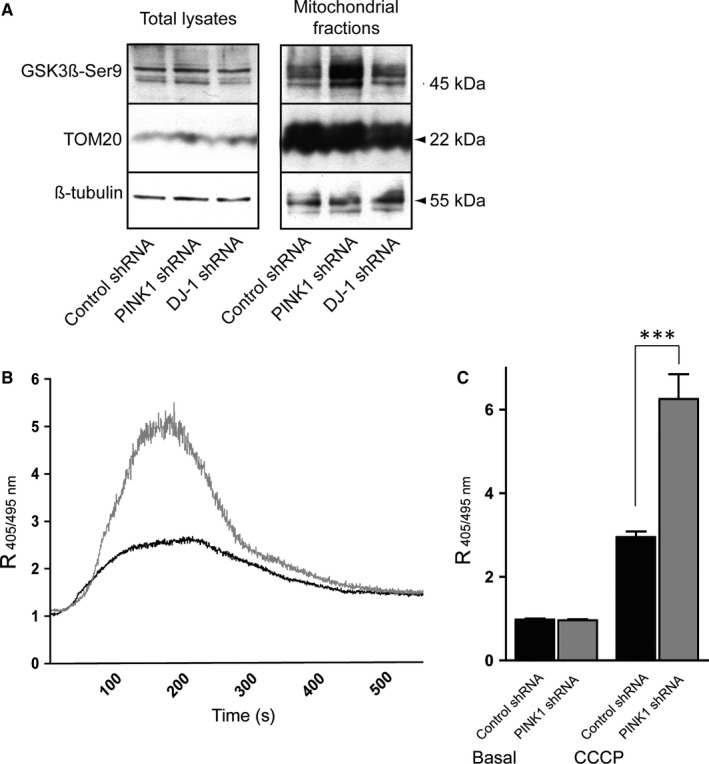
Mitochondria in PINK1 deficient cells contain increased levels of Ser9 phosphorylated GSK3β and release more Ca^2+^ into the cytosol when exposed to CCCP compared to control shRNA. Western blot of total (whole cell lysate) and mitochondrial fractions obtained from control, PINK1 and DJ‐1 knockdown cell lines probed for GSK3βSer9, TOM20, DJ‐1, and β‐tubulin. Mitochondrial fractions in PINK1 knockdown cells contain higher levels of GSK3βSer9 compared to control and DJ‐1 knockdown cells (n = 3) (A). Effect of the mitochondrial uncoupler CCCP on [Ca^2+^]_c_ in Control and PINK1 shRNA cells expressed as 405/495 ratio. Data show one representative experiment of each cell line superimposed (black: control shRNA, grey: PINK1 shRNA) (B). Quantification of [Ca^2+^]_c_ in resting cells and after mitochondrial depolarization by 1 μmol/L CCCP. Maximum levels after CCCP exposure were determined by taking the mean from 10 s at the highest 405/495 peak (C). Data expressed as means ± SEM of ratio between the 405 and 495 nm emissions of six independent experiments using Mann‐Whitney test (***P < 0.001)

In an attempt to search for a common mechanism linking deficient motility with PD mutations, we investigated if proteins relevant for calcium and mitochondrial motility was altered in mitochondrial fractions from PINK1 or DJ‐1 depleted cells. Glycogen synthase kinase‐3β (GSK3β) is a key serine/threonine protein kinase for multiple mitochondrial functions such as biogenesis, bioenergetics, permeability and motility (for review, see Yang et al^45^). We evaluated whether the loss of PINK1 and DJ‐1 may modulate GSK3β activity in whole cell lysates (total fractions) and mitochondrial fractions using Western blot analyses. As shown in Figure 3A, PINK1 deficient cells had increased levels of inhibitory phosphorylation of GSK‐3β in the mitochondrial fraction, whereas total lysates showed no differences among cell lines. No changes in GSK3β‐Ser9 levels were seen in the mitochondrial fraction of DJ‐1 shRNA cells. TOM20 (mitochondrial import receptor subunit) and β‐tubulin levels were used as control for mitochondrial purification and loading for total fractions, respectively. These data indicate that loss of PINK1 leads to an inhibitory state of GSK‐3β at the mitochondrial compartments by phosphorylation of serine‐9.

### 3.4 Increase of mitochondrial Ca^2+^ efflux in PINK deficient cells exposed to CCCP

Previous studies in other models show that PINK1 downregulation leads to an accumulation of calcium in the mitochondria.^46^, ^47^ As a result of the fact that GSK3β Ser9 levels has been shown to block the permeability trasition pore and thus impair calcium buffering we investigated whether the increased levels of GSK‐3βSer9 found in our PINK deficient cells model may influence mitochondrial Ca^2+^ efflux. The ratiometric dye Indo‐1‐AM is excited with light at 360 ± 5 nm. Whether light is emitted at 405 ± 5 nm represents the Ca^2+^ bound Indo‐1‐AM; on the contrary, emission at 495 ± 5 nm represents the Ca^2+^ free Indo‐1‐AM. Spectra were registered every 0.5 second after 10 seconds of incubation. Figure 3B show the ratio of Indo‐1 signals (R_405/495_) in PINK shRNA cells and control shRNA cells exposed to 1 μmol/L CCCP (mitochondrial uncoupling agent). As shown in Figure 3C, an increase in 405 nm simultaneously followed by a decrease in 495 nm emission in PINK1 knockdown cells resulted in a significantly higher intracellular release of Ca^2+^ after uncoupling of mitochondria with CCCP compared to control (*P* < 0.001).

### 3.5 Downregulation of PINK1 or DJ‐1 proteins results in impaired connectivity between ER and mitochondria

**Figure 4:**
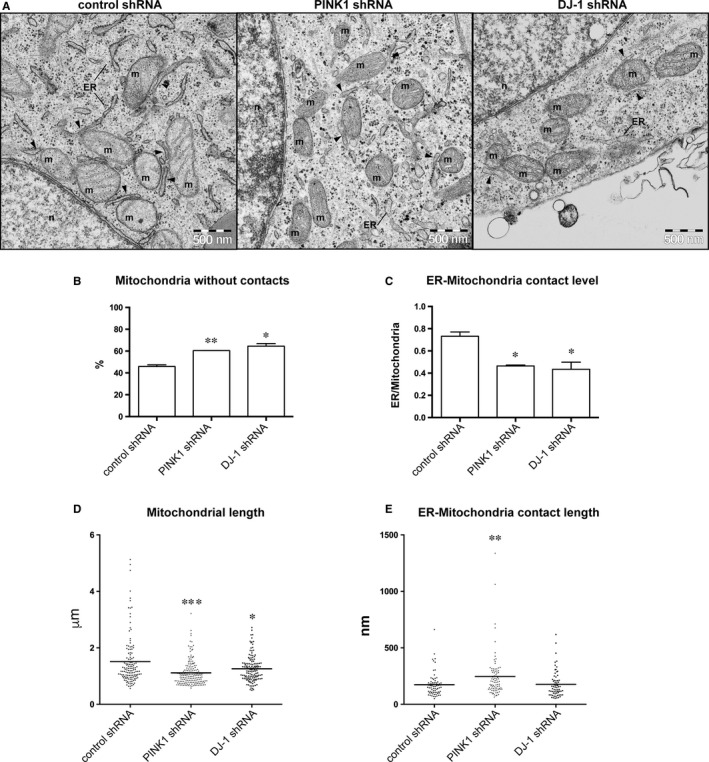
Downregulation of PINK1 or DJ‐1 proteins results in impaired connectivity between ER and mitochondria. Transmission electron microscopy (TEM) micrographs of ER‐mitochondria contacts (shown by black arrowheads) in control, PINK1 and DJ1 shRNA cells (m: mitochondria, n: nucleus, ER: endoplasmic reticulum) (A). Quantification of TEM micrographs show that cells expressing PINK1 and DJ‐1 shRNA have a higher number of mitochondria without contacts with ER compared to control shRNA (B). PINK1 and DJ‐1 shRNA cells have lower number of ER‐mitochondria contacts as quantified from TEM and normalized to the total number of mitochondria for each cell line (C). Mitochondrial length is significantly reduced in PINK1 and DJ‐1 undifferentiated cells (D). The mean length of each contact was found to be significantly increased in PINK1 shRNA cells compared to control shRNA (E). Data expressed as means ± SEM using Mann‐Whitney test (*P < 0.05, **P < 0.01, ***P < 0.001)

The elevated mitochondrial calcium release seen in PINK1 deficient cells proposes that there is an imbalance in the calcium handling. The possible explanations to this imbalance may be that PINK1 knockdown impair cytosolic calcium buffering via the uniporter, or that there is an imbalance in the ER‐mitochondria calcium shuttling system. Basal cytosolic calcium was not altered in the PINK1 knockdown cells. Therefore, we thought that the calcium shuttle from ER may be impaired in these cells. Electron microscopy images of control, PINK1 and DJ‐1 shRNA undifferentiated cells were quantified with regards to the number and length of ER‐mitochondria contacts. PINK1 and DJ‐1 deficient cells showed higher number of mitochondria without ER‐contacts (Figure 4A) and significantly lower number of ER‐mitochondria contacts (Figure 4B). The mitochondrial length quantified in the EM micrographs was significantly shorter in PINK1 and DJ‐1 shRNA cells than in control cells (Figure 4C), however the length of ER‐mitochondria contacts was significantly longer in PINK1 shRNA cells (Figure 4D).

## 4 DISCUSSION

Mitochondrial tubular anchorage and transport are necessary for mitochondrial fusion^48^ and it has been reported that the retrograde transport motor dynein can inhibit mitochondrial fission and redistribute mitochondria from the axon to the soma.^49^ Thus, there are important links between mitochondrial dynamics and transport. We and others have previously reported a decreased rate of fusion in human PINK1 and DJ‐1 knockdown cells and that this phenotype can be reversed by downregulating the fission protein Drp1.^25^, ^36^, ^37^, ^38^ In the current study, we investigate how PINK1 or DJ‐1 knockdown affect mitochondrial transport in a human, RA/BDNF differentiated neuroblastoma model. Simultaneously, we explored the potential consequences of PINK1/Drp1 or DJ‐1/Drp1 double knockdown.

In our hands, we did not detect any differences in the size or distribution of mitochondria along the neurites when downregulating PINK1 or DJ‐1, neither was there any effect on dendrite outgrowth nor were there differences in any of the above mentioned aspects for the double knockdown with Drp1 siRNA. In line with this finding, another research group has shown that both Drp1 protein levels and activity do not alter neurite outgrowth in PINK1 knockdown cells.^44^

In contrast with the unaffected neuritic mitochondrial length, undifferentiated cells lacking PINK1 or DJ‐1 had significantly shorter mitochondria compared to control which is in line with previous data from us and others.^25^, ^38^ This compartmental difference between cell body and axons was also observed in a recent *in vivo* study by Devireddy et al^41^ where the authors demonstrate that PINK1 regulates mitochondrial motility in axons and mitochondrial morphology in the cell soma, but not fusion or turnover in axons of mature neurons. It is likely that this difference in mitochondrial length between cell body and neurite seen by us and others could be a consequence from mitochondrial turnover mostly occurring in the soma.^50^

The status of the mitochondria determines their bidirectional transport along the neurites. Intracellular conditions such as local energetic demands and Ca^2+^ levels influence the transport of mitochondria.^51^ Mitochondrial membrane potential (ΔΨ_m_) is determining the rate and direction of transport, where low potential favours retrograde transport and high potential anterograde transport.^52^ Since ΔΨ_m_ has been shown to be decreased in cells lacking PINK1^8^, ^41^ and DJ‐1 knockdown,^25^ an impairment of anterograde transport would be a coherent downstream consequence. Yet, other factors such as calcium levels and the PINK1 interacting protein Miro1 also has important roles for mitochondrial motility. Previous reports from the role of PINK1 on motility have been ambiguous, showing that knockdown either stimulate or impair mitochondrial transport.^15^, ^17^, ^41^ It appears that our study supports the latter mechanism, demonstrating that loss of PINK1 impedes the mitochondrial trafficking in both directions and the same trend was seen in DJ‐1 knockdown cells. Differences in experimental models or setups including *in vitro* or *in vivo* are likely to explain this disparity between studies^39^ since the same conflicting data are seen on the role of PINK1 in mitochondrial dynamics.^36^, ^37^, ^53^ Further investigations should focus on identifying why the role of PINK1 may differ between models and cells, as this may be of relevance in the search for effective targeted PD therapies.

Active mitochondria are more prone to undergo anterograde transport, a feature that has been proposed to be induced by the cleavage and phosphorylation of PINK1^9^, ^54^ in association to adaptor proteins Miro/Milton and Kinesin motors. PINK1 has been shown by us and others to interact with and mediate degradation of Miro1.^19^ As a result of the central role of Miro1 in mitochondrial trafficking, we measured its levels in PINK1 and DJ1 knockdown cells. Interestingly, Miro1 levels were found to be significantly higher in PINK1 depleted cells, suggesting that the impaired mitochondrial transport seen in our cells involve Miro1. Indeed, other reports have shown that overexpressing Miro1 impair mitochondrial transport in a calcium‐dependent manner.^55^, ^56^

In contrast to the suppressive effect of Drp1 knockdown on the induced‐mitochondrial fragmentation by PINK1 gene silencing,^38^ we found that co‐downregulation of Drp1 resulted in a slight increase in the number of motile events in both PINK1 or DJ‐1 knockdown cells. This restrains the discrepancy between knockdown and control cells but nevertheless the statistical variability of the results observed in double knockdown cells does not clearly define a rescue effect *per se* on mitochondrial motility or mitochondrial density by inhibiting Drp1‐mediated mitochondrial fission. These findings are in line with a previous study where neither wild‐type Drp1 or a PKA phosphor‐mimetic mutant of Drp1 (S656D) had any effect on mitochondrial movement or density in dendrites of mouse PINK1 deficient neurons.^44^

The motor protein kinesin, involved in the anterograde transport towards the synapse is regulated by the serine/threonine kinase GSK3β.^57^ GSK3β, a multifunctional kinase regulating more than 40 different substrates, is regulated by phosphorylation of Serine9 (inactivation) or Tyrosine216 (activation)^58^ by pro‐survival kinases such as Akt, Protein kinase C‐ε (PKCε), extracellular signal regulated kinase (ERK), and protein kinase G. Moreover, GSK3 is a central protein for multiple mitochondrial functions including motility (for review see Ref. 45). We found that the inactivated GSK3βSer9 accumulates in mitochondrial fractions of PINK1 but not DJ‐1 knockdown cells. However, we did not detect any changes in the protein levels of GSK3βTyr216 or PKCε (data not shown). GSK3βSer9 has formerly been demonstrated to inhibit the mitochondrial permeability transition pore (mPTP),^59^ which in light of our findings would imply that the mPTP of PINK1 knockdown results in an increased threshold for pore opening compared to control cells. Since the mPTP is important in regulating mitochondrial Ca^2+^ load we measured cytosolic Ca^2+^ before and after treatment with the mitochondrial uncoupler, CCCP. The basal levels of Ca^2+^ did not differ between PINK1 deficient and control cells; however CCCP‐mediated mitochondrial uncoupling led to a more dramatic increase in cytosolic Ca^2+^ levels in PINK1 knockdown background. One possibility is that the difference arises from an elevated mitochondrial Ca^2+^ load because of GSK3βSer9‐mediated blocking in the mPTP of PINK1 knockdown cells. Indeed previous studies have showed similarly to our data that mitochondria in PINK1 knock‐out cells contain higher Ca^2+^ levels and release more cytosolic Ca^2+^ upon mitochondrial uncoupling.^60^, ^61^ Since adult dopaminergic neurons typically depend on Ca^2+^ channels instead of Na^+^ channels for generating action potentials,^62^ the mitochondrial Ca^2+^ buffering in this group of neurons plays a major role in response to mitochondrial stress.

The fact that the buffering capacity was not impaired in PINK1 deficient cells at basal level may be linked to a compensatory effect from the ER. Since Ca^2+^ can be transferred from ER to mitochondria at the contact sites, we investigated the connectivity between both organelles. Interestingly, both PINK1 and DJ‐1 knockdown cells showed a decreased number of ER‐mitochondria contact sites as compared to control cells. This finding is in agreement with previous reports showing that overexpression of Parkin or DJ‐1 result in increased connectivity between ER and mitochondria.^32^, ^33^ Yet, the mean contact length was significantly increased in PINK1 but not DJ‐1 knockdown cells.

Our results suggests that PINK1 and DJ‐1 are important for the fusion between ER and mitochondria. Whether the increased ER‐mitochondria contact length seen in PINK1 deficient cells compensate for the decreased number of ER‐mitochondria contact sites remains to be clarified. Indeed, more studies are needed to clarify how PD gene mutations alter the ER‐mitochondria contacts. Taken all together, our results demonstrate that the frequency of mitochondrial movement in the neurites of differentiated cells is reduced as a result of either PINK1 or DJ‐1 lack of function that also have an impact in the ER‐mitochondria apposition. This could be one of the common mechanisms by which mutations in PINK1 or DJ‐1 contribute to neurodegeneration in autosomal recessive parkinsonism.

## CONFLICT OF INTEREST

The authors declare no conflict of interest.

## References

[1] Lin MT , Beal MF . Mitochondrial dysfunction and oxidative stress in neurodegenerative diseases. Nature. 2006;443:787‐795. 17051205 10.1038/nature05292

[2] Schapira AH . Mitochondria in the aetiology and pathogenesis of Parkinson's disease. Lancet Neurol. 2008;7:97‐109. 18093566 10.1016/S1474-4422(07)70327-7

[3] Vila M , Ramonet D , Perier C . Mitochondrial alterations in Parkinson's disease: new clues. J Neurochem. 2008;107:317‐328. 18680555 10.1111/j.1471-4159.2008.05604.x

[4] Celsi F , Pizzo P , Brini M , et al. Mitochondria, calcium and cell death: a deadly triad in neurodegeneration. Biochim Biophys Acta. 2009;1787:335‐344. 19268425 10.1016/j.bbabio.2009.02.021 PMC2696196

[5] Soubannier V , McBride HM . Positioning mitochondrial plasticity within cellular signaling cascades. Biochim Biophys Acta. 2009;1793:154‐170. 18694785 10.1016/j.bbamcr.2008.07.008

[6] Huang E , Qu D , Huang T , et al. PINK1‐mediated phosphorylation of LETM1 regulates mitochondrial calcium transport and protects neurons against mitochondrial stress. Nat Commun. 2017;8:1399. 29123128 10.1038/s41467-017-01435-1 PMC5680261

[7] Ashrafi G , Schwarz TL . The pathways of mitophagy for quality control and clearance of mitochondria. Cell Death Differ. 2013;20:31‐42. 22743996 10.1038/cdd.2012.81 PMC3524633

[8] Exner N , Treske B , Paquet D , et al. Loss‐of‐function of human PINK1 results in mitochondrial pathology and can be rescued by parkin. J Neurosci. 2007;27:12413‐12418. 17989306 10.1523/JNEUROSCI.0719-07.2007 PMC6673250

[9] Narendra DP , Jin SM , Tanaka A , et al. PINK1 is selectively stabilized on impaired mitochondria to activate Parkin. PLoS Biol. 2010;8:e1000298. 20126261 10.1371/journal.pbio.1000298 PMC2811155

[10] Yang Y , Gehrke S , Imai Y , et al. Mitochondrial pathology and muscle and dopaminergic neuron degeneration caused by inactivation of Drosophila Pink1 is rescued by Parkin. Proc Natl Acad Sci USA. 2006;103:10793‐10798. 16818890 10.1073/pnas.0602493103 PMC1502310

[11] Beilina A , Van Der Brug M , Ahmad R , et al. Mutations in PTEN‐induced putative kinase 1 associated with recessive parkinsonism have differential effects on protein stability. Proc Natl Acad Sci USA. 2005;102:5703‐5708. 15824318 10.1073/pnas.0500617102 PMC556294

[12] Lin W , Kang UJ . Characterization of PINK1 processing, stability, and subcellular localization. J Neurochem. 2008;106:464‐474. 18397367 10.1111/j.1471-4159.2008.05398.x PMC3638740

[13] Pridgeon JW , Olzmann JA , Chin LS , Li L . PINK1 protects against oxidative stress by phosphorylating mitochondrial chaperone TRAP1. PLoS Biol. 2007;5:e172. 17579517 10.1371/journal.pbio.0050172 PMC1892574

[14] Silvestri L , Caputo V , Bellacchio E , et al. Mitochondrial import and enzymatic activity of PINK1 mutants associated to recessive parkinsonism. Hum Mol Genet. 2005;14:3477‐3492. 16207731 10.1093/hmg/ddi377

[15] Shlevkov E , Kramer T , Schapansky J , LaVoie MJ , Schwarz TL . Miro phosphorylation sites regulate Parkin recruitment and mitochondrial motility. Proc Natl Acad Sci USA. 2016;113:E6097‐E6106. 27679849 10.1073/pnas.1612283113 PMC5068282

[16] Gelmetti V , De Rosa P , Torosantucci L , et al. PINK1 and BECN1 relocalize at mitochondria‐associated membranes during mitophagy and promote ER‐mitochondria tethering and autophagosome formation. Autophagy. 2017;13:654‐669. 28368777 10.1080/15548627.2016.1277309 PMC5388214

[17] Liu S , Sawada T , Lee S , et al. Parkinson's disease‐associated kinase PINK1 regulates Miro protein level and axonal transport of mitochondria. PLoS Genet. 2012;8:e1002537. 22396657 10.1371/journal.pgen.1002537 PMC3291531

[18] Wang X , Winter D , Ashrafi G , et al. PINK1 and Parkin target Miro for phosphorylation and degradation to arrest mitochondrial motility. Cell. 2011;147:893‐906. 22078885 10.1016/j.cell.2011.10.018 PMC3261796

[19] Weihofen A , Thomas KJ , Ostaszewski BL , Cookson MR , Selkoe DJ . Pink1 forms a multiprotein complex with Miro and Milton, linking Pink1 function to mitochondrial trafficking. Biochemistry. 2009;48:2045‐2052. 19152501 10.1021/bi8019178 PMC2693257

[20] Paillusson S , Stoica R , Gomez‐Suaga P , et al. There's something wrong with my MAM; the ER‐mitochondria axis and neurodegenerative diseases. Trends Neurosci. 2016;39:146‐157. 26899735 10.1016/j.tins.2016.01.008 PMC4780428

[21] Erpapazoglou Z , Mouton‐Liger F , Corti O . From dysfunctional endoplasmic reticulum‐mitochondria coupling to neurodegeneration. Neurochem Int. 2017;109:171–183. 28389271 10.1016/j.neuint.2017.03.021

[22] Bonifati V , Rizzu P , van Baren MJ , et al. Mutations in the DJ‐1 gene associated with autosomal recessive early‐onset parkinsonism. Science. 2003;299:256‐259. 12446870 10.1126/science.1077209

[23] Requejo‐Aguilar R , Lopez‐Fabuel I , Jimenez‐Blasco D , Fernandez E , Almeida A , Bolaños JP . DJ1 represses glycolysis and cell proliferation by transcriptionally up‐regulating Pink1. Biochem J. 2015;467:303‐310. 25670069 10.1042/BJ20141025 PMC4385472

[24] Kim RH , Peters M , Jang Y , et al. DJ‐1, a novel regulator of the tumor suppressor PTEN. Cancer Cell. 2005;7:263‐273. 15766664 10.1016/j.ccr.2005.02.010

[25] Thomas KJ , McCoy MK , Blackinton J , et al. DJ‐1 acts in parallel to the PINK1/parkin pathway to control mitochondrial function and autophagy. Hum Mol Genet. 2011;20:40‐50. 20940149 10.1093/hmg/ddq430 PMC3000675

[26] van der Brug MP , Blackinton J , Chandran J , et al. RNA binding activity of the recessive parkinsonism protein DJ‐1 supports involvement in multiple cellular pathways. Proc Natl Acad Sci USA. 2008;105:10244‐10249. 18626009 10.1073/pnas.0708518105 PMC2481328

[27] Yang Y , Gehrke S , Haque ME , et al. Inactivation of Drosophila DJ‐1 leads to impairments of oxidative stress response and phosphatidylinositol 3‐kinase/Akt signaling. Proc Natl Acad Sci USA. 2005;102:13670‐13675. 16155123 10.1073/pnas.0504610102 PMC1224636

[28] Canet‐Aviles RM , Wilson MA , Miller DW , et al. The Parkinson's disease protein DJ‐1 is neuroprotective due to cysteine‐sulfinic acid‐driven mitochondrial localization. Proc Natl Acad Sci USA. 2004;101:9103‐9108. 15181200 10.1073/pnas.0402959101 PMC428480

[29] Zhang L , Shimoji M , Thomas B , et al. Mitochondrial localization of the Parkinson's disease related protein DJ‐1: implications for pathogenesis. Hum Mol Genet. 2005;14:2063‐2073. 15944198 10.1093/hmg/ddi211

[30] Junn E , Jang WH , Zhao X , Jeong BS , Mouradian MM . Mitochondrial localization of DJ‐1 leads to enhanced neuroprotection. J Neurosci Res. 2009;87:123‐129. 18711745 10.1002/jnr.21831 PMC2752655

[31] Taira T , Saito Y , Niki T , Iguchi‐Ariga SM , Takahashi K , Ariga H . DJ‐1 has a role in antioxidative stress to prevent cell death. EMBO Rep. 2004;5:213‐218. 14749723 10.1038/sj.embor.7400074 PMC1298985

[32] Cali T , Ottolini D , Negro A , Brini M . Enhanced parkin levels favor ER‐mitochondria crosstalk and guarantee Ca(2+) transfer to sustain cell bioenergetics. Biochim Biophys Acta. 2013;1832:495‐508. 23313576 10.1016/j.bbadis.2013.01.004

[33] Ottolini D , Cali T , Negro A , Brini M . The Parkinson disease‐related protein DJ‐1 counteracts mitochondrial impairment induced by the tumour suppressor protein p53 by enhancing endoplasmic reticulum‐mitochondria tethering. Hum Mol Genet. 2013;22:2152‐2168. 23418303 10.1093/hmg/ddt068

[34] Krebiehl G , Ruckerbauer S , Burbulla LF , et al. Reduced basal autophagy and impaired mitochondrial dynamics due to loss of Parkinson's disease‐associated protein DJ‐1. PLoS ONE. 2010;5:e9367. 20186336 10.1371/journal.pone.0009367 PMC2826413

[35] Chan DC . Mitochondrial fusion and fission in mammals. Annu Rev Cell Dev Biol. 2006;22:79‐99. 16704336 10.1146/annurev.cellbio.22.010305.104638

[36] Dagda RK , Cherra SJ 3rd , Kulich SM , Tandon A , Park D , Chu CT . Loss of PINK1 function promotes mitophagy through effects on oxidative stress and mitochondrial fission. J Biol Chem. 2009;284:13843‐13855. 19279012 10.1074/jbc.M808515200 PMC2679485

[37] Lutz AK , Exner N , Fett ME , et al. Loss of parkin or PINK1 function increases Drp1‐dependent mitochondrial fragmentation. J Biol Chem. 2009;284:22938‐22951. 19546216 10.1074/jbc.M109.035774 PMC2755701

[38] Sandebring A , Thomas KJ , Beilina A , et al. Mitochondrial alterations in PINK1 deficient cells are influenced by calcineurin‐dependent dephosphorylation of dynamin‐related protein 1. PLoS ONE. 2009;4:e5701. 19492085 10.1371/journal.pone.0005701 PMC2683574

[39] Grenier K , McLelland GL , Fon EA . Parkin‐ and PINK1‐dependent mitophagy in neurons: will the real pathway please stand up? Front. Neurol. 2013;4:100. 23882257 10.3389/fneur.2013.00100 PMC3715719

[40] Sulzer D , Surmeier DJ . Neuronal vulnerability, pathogenesis, and Parkinson's disease. Mov Disord. 2013 Jun;28(6):715‐724. 23589357 10.1002/mds.25187

[41] Devireddy S , Liu A , Lampe T , Hollenbeck PJ . The organization of mitochondrial quality control and life cycle in the nervous system in vivo in the absence of PINK1. J Neurosci. 2015;35:9391‐9401. 26109662 10.1523/JNEUROSCI.1198-15.2015 PMC4478254

[42] Schreiner B , Ankarcrona M . Isolation of mitochondria‐associated membranes (MAM) from mouse brain tissue. Methods Mol Biol. 2017;1567:53‐68. 28276013 10.1007/978-1-4939-6824-4_5

[43] Kocsis E , Trus BL , Steer CJ , Bisher ME , Steven AC . Image averaging of flexible fibrous macromolecules: the clathrin triskelion has an elastic proximal segment. J Struct Biol. 1991;107:6‐14. 1817611 10.1016/1047-8477(91)90025-r

[44] Das Banerjee T , Dagda RY , Dagda M , et al. PINK1 regulates mitochondrial trafficking in dendrites of cortical neurons through mitochondrial PKA. J Neurochem. 2017;142:545‐559. 28556983 10.1111/jnc.14083 PMC5554084

[45] Yang K , Chen Z , Gao J , et al. The key roles of GSK‐3β in regulating mitochondrial activity. Cell Physiol Biochem. 2017;44:1445‐1459. 29190615 10.1159/000485580

[46] Deas E , Plun‐Favreau H , Wood NW . PINK1 function in health and disease. EMBO Mol Med. 2009;1:152‐165. 20049715 10.1002/emmm.200900024 PMC3378127

[47] Soman S , Keatinge M , Moein M , et al. Inhibition of the mitochondrial calcium uniporter rescues dopaminergic neurons in pink1−/− zebrafish. Eur J Neurosci. 2017;45:528‐535. 27859782 10.1111/ejn.13473 PMC5324670

[48] Liu X , Weaver D , Shirihai O , Hajnóczky G . Mitochondrial ‘kiss‐and‐run’: interplay between mitochondrial motility and fusion‐fission dynamics. EMBO J. 2009;28:3074‐3089. 19745815 10.1038/emboj.2009.255 PMC2771091

[49] Varadi A , Johnson‐Cadwell LI , Cirulli V , Yoon Y , Allan VJ , Rutter GA . Cytoplasmic dynein regulates the subcellular distribution of mitochondria by controlling the recruitment of the fission factor dynamin‐related protein‐1. J Cell Sci. 2004;117:4389‐4400. 15304525 10.1242/jcs.01299

[50] Cai Q , Zakaria HM , Simone A , Sheng ZH . Spatial parkin translocation and degradation of damaged mitochondria via mitophagy in live cortical neurons. Curr Biol. 2012;22:545‐552. 22342752 10.1016/j.cub.2012.02.005 PMC3313683

[51] Jeyaraju DV , Cisbani G , Pellegrini L . Calcium regulation of mitochondria motility and morphology. Biochim Biophys Acta. 2009;1787:1363‐1373. 19138660 10.1016/j.bbabio.2008.12.005

[52] Miller KE , Sheetz MP . Axonal mitochondrial transport and potential are correlated. J Cell Sci. 2004;117:2791‐2804. 15150321 10.1242/jcs.01130

[53] Yang Y , Ouyang Y , Yang L , et al. Pink1 regulates mitochondrial dynamics through interaction with the fission/fusion machinery. Proc Natl Acad Sci USA. 2008;105:7070‐7075. 18443288 10.1073/pnas.0711845105 PMC2383971

[54] Matenia D , Hempp C , Timm T , Eikhof A , Mandelkow EM . Microtubule affinity‐regulating kinase 2 (MARK2) turns on phosphatase and tensin homolog (PTEN)‐induced kinase 1 (PINK1) at Thr‐313, a mutation site in Parkinson disease: effects on mitochondrial transport. J Biol Chem. 2012;287:8174‐8186. 22238344 10.1074/jbc.M111.262287 PMC3318756

[55] Saotome M , Safiulina D , Szabadkai G , et al. Bidirectional Ca^2+^‐dependent control of mitochondrial dynamics by the Miro GTPase. Proc Natl Acad Sci USA. 2008;105:20728‐20733. 19098100 10.1073/pnas.0808953105 PMC2634948

[56] Russo GJ , Louie K , Wellington A , et al. Drosophila Miro is required for both anterograde and retrograde axonal mitochondria transport. J Neurosci. 2009;29:5443‐5455. 19403812 10.1523/JNEUROSCI.5417-08.2009 PMC2693725

[57] Morfini G , Szebenyi G , Elluru R , Ratner N , Brady ST . Glycogen synthase kinase 3 phosphorylates kinesin light chains and negatively regulates kinesin‐based motility. EMBO J. 2002;21:281‐293. 11823421 10.1093/emboj/21.3.281 PMC125832

[58] Song JS , Yang SD . Tau protein kinase I/GSK‐3 beta/kinase FA in heparin phosphorylates tau on Ser199, Thr231, Ser235, Ser262, Ser369, and Ser400 sites phosphorylated in Alzheimer disease brain. J Protein Chem. 1995;14:95‐105. 7786411 10.1007/BF01888367

[59] Juhaszova M , Zorov DB , Kim SH , et al. Glycogen synthase kinase‐3beta mediates convergence of protection signaling to inhibit the mitochondrial permeability transition pore. J Clin Invest. 2004;113:1535‐1549. 15173880 10.1172/JCI19906 PMC419483

[60] Gautier CA , Giaime E , Caballero E , et al. Regulation of mitochondrial permeability transition pore by PINK1. Mol Neurodegener. 2012;7:22. 22630785 10.1186/1750-1326-7-22 PMC3405481

[61] Gandhi S , Wood‐Kaczmar A , Yao Z , et al. PINK1‐associated Parkinson's disease is caused by neuronal vulnerability to calcium‐induced cell death. Mol Cell. 2009;33:627‐638. 19285945 10.1016/j.molcel.2009.02.013 PMC2724101

[62] Chan CS , Guzman JN , Ilijic E , et al. ‘Rejuvenation’ protects neurons in mouse models of Parkinson's disease. Nature. 2007;447:1081‐1086. 17558391 10.1038/nature05865

