# Corrigendum

**DOI:** 10.1111/jcmm.14222

**Published:** 2019-05-23

Reduction of PINK1 or DJ‐1 impair mitochondrial motility in neurites and alter ER‐mitochondria contacts

Cristina Parrado‐Fernández | Bernadette Schreiner | Maria Ankarcrona | Melissa M. Conti | Mark R. Cookson | Miia Kivipelto | Ángel Cedazo‐Mínguez | Anna Sandebring‐Matton

In the above article, the surname for the second author was incorrectly spelt in the published version. The correct name should be Bernadette Schreiner. The online version has been corrected.

The author apologize for the inconvenience caused.
